# A billion-dollar donation: estimating the cost of researchers’ time spent on peer review

**DOI:** 10.1186/s41073-021-00118-2

**Published:** 2021-11-14

**Authors:** Balazs Aczel, Barnabas Szaszi, Alex O. Holcombe

**Affiliations:** 1 Present address: Institute of Psychology, ELTE, Eotvos Lorand University, Izabella u. 46, Budapest, 1064 Hungary; 2 School of Psychology, University of Sydney, Sydney, Australia

**Keywords:** Peer-review, Academic publishers, Publication system

## Abstract

**Background:**

The amount and value of researchers’ peer review work is critical for academia and journal publishing. However, this labor is under-recognized, its magnitude is unknown, and alternative ways of organizing peer review labor are rarely considered.

**Methods:**

Using publicly available data, we provide an estimate of researchers’ time and the salary-based contribution to the journal peer review system.

**Results:**

We found that the total time reviewers globally worked on peer reviews was over 100 million hours in 2020, equivalent to over 15 thousand years. The estimated monetary value of the time US-based reviewers spent on reviews was over 1.5 billion USD in 2020. For China-based reviewers, the estimate is over 600 million USD, and for UK-based, close to 400 million USD.

**Conclusions:**

By design, our results are very likely to be under-estimates as they reflect only a portion of the total number of journals worldwide. The numbers highlight the enormous amount of work and time that researchers provide to the publication system, and the importance of considering alternative ways of structuring, and paying for, peer review. We foster this process by discussing some alternative models that aim to boost the benefits of peer review, thus improving its cost-benefit ratio.

## Background

One of the main products of the academic publication system, the journal article, is a co-production of researchers and publishers. Researchers provide value not only by doing the research and writing up the results as a manuscript, but also by serving as peer reviewers. Publishers provide services of selection, screening, and dissemination of articles, including ensuring (proper) meta-data indexing in databases. Although several careful estimates are available regarding the cost of academic publishing e.g., [1], one aspect these estimates often neglect is the cost of peer reviews [2]. Our aim was to provide a timely estimation of reviewers’ contribution to the publication system in terms of time and financial value and discuss the implications.

In their peer reviewer role, scientists and other researchers provide comments to improve other researchers’ manuscripts and judge their quality. They offer their time and highly specialized knowledge to provide a detailed evaluation and suggestions for improvement of manuscripts. On average, a reviewer completes 4.73 reviews per year,^1^ yet, according to Publons,^2^ certain reviewers complete over a thousand reviews a year. This contribution takes considerable time from other academic work. In the biomedical domain alone, the time devoted to peer review in 2015 was estimated to be 63.4 M hours [3].

A manuscript typically receives multiple rounds of reviews before acceptance, and each round typically involves two or more researchers as peer reviewers. Peer review work is rarely formally recognized or directly financially compensated in the journal system (exceptions include some medical journals that pay for statistical reviewers and some finance journals that pay for quick referee reports). Most universities seem to expect academics to do review work as part of their research or scholarly service mission, although we know of none with an explicit policy about how much time they should spend on it.

While peer review work is a critical element of academic publishing, we found only a single estimate of its financial value, which was from 2007. Then, when the global number of published articles was not even half of the present volume, rough estimates indicated that if reviewers were paid for their time, the bill would be on the order of £1.9bn [4].

As a facet of the research process that currently requires labor by multiple human experts, reviewing contributes to a cost disease situation for science. “Cost disease” [5] refers to the fact that while the cost of many products and services have steadily decreased over the last two hundred years, this has not happened for some for which the amount of labor time per unit has not changed. This can make some products and services increasingly expensive relative to everything else in society, as has occurred, for example, for live classical music concerts. This may also be the fate of scholarly publication, unless reviewing is made more efficient.

The fairness and efficiency of the traditional peer review system has recently become a highly-debated topic [6, 7]. In this paper, we extend this discussion by providing an update on the estimate of researchers’ time and the salary-based contribution to the peer-review system. We used publicly available data for our calculations. Our approximation is almost certainly an underestimate because not only do we choose conservative values of parameters, but for the total number of academic articles, we rely on a database (Dimensions) that does not purport to include every journal in the world. We discuss the implications of our estimates and identify a number of alternative models for better utilizing research time in peer review.

## Methods and results

To estimate the time and the salary-based monetary value of the peer review conducted for journals in a single year, we had to estimate the number of peer reviews per year, the average time spent per review, and the hourly labor cost of academics. In case of uncertainty, we used conservative estimates for our parameters, therefore, the true values are likely to be higher.

### Coverage

The total number of articles is obviously a critical input for our calculation. Unfortunately, there appears to be no database available that includes all the academic articles published in the entire world. Ulrich’s Periodicals Database may list the largest number of journals - querying their database for “journals” or “conference proceedings” and “Refereed / Peer-reviewed” yielded 99,753 entries. However, Ulrich’s does not indicate the number of articles that these entities publish. Out of the available databases that do report the number of articles, we chose to use Dimensions’ dataset (https://www.dimensions.ai/) which collects and collected articles from 87,000 scholarly journals, much more than Scopus (~ 20,000) or Web of Science (~ 14,000) [8].

### Number of peer reviews per year

Only estimates exist for how many peer reviews associated with journals occur each year. Publons [9] estimated that the 2.9 million articles indexed in the Web of Science in 2016 required 13.7 million reviews. To calculate the number of reviews relevant to 2020, we used the formula used by Publons [9] - eq. 1 below. In that formula, a review is what *one* researcher does in *one* round of a review process.^3^ For submissions that are ultimately accepted by the journal submitted to, the Publons formula assumes that on average there are two reviews in the first round and one in the second round; for rejected articles (excluding desk rejections) the formula assumes an average of two reviews for submissions that are ultimately rejected, both in the first round. Publons’ assumptions are based on their general knowledge of the industry but no specific data. Note, however, that if anything these are most likely underestimations as not all peer reviews are included in our estimation. For example, the review work done by some editors when handling a manuscript is not usually indexed in Publons, and a single written review report may be signed by several researchers.

Publons estimated the acceptance rate for peer-reviewed submissions to be 55%. That is, 45% of manuscripts that are not desk rejected are, after one or more rounds of review, ultimately rejected. Before including Publons’ estimates in our calculations, we evaluated them based on other available information. The Thomson Reuters publishing company reported numbers regarding the submissions, acceptances, and rejections that occurred at their ScholarOne journal management system for the period 2005–2010 [10]. In agreement with other sources [11, 12], it showed that the mean acceptance rates have apparently declined [10], the proportion of submissions that are eventually accepted by the journal the manuscript was submitted at was 0.40 in 2005: 0.37 in 2010, and 0.35 in 2011 [11, 12].

We did not find estimates of acceptance rates for the last several years, but we assume that the decline described by Thomson Reuters [10] continued to some extent, and assume that the present mean acceptance rate at journals is 0.30 then we can arrive at Publons’ figures. However, for the final numbers, we also need to estimate the rate of desk rejections as well. Although the rate of desk rejections likely varies substantially across journals (e.g., 22–26% at PLOS ONE^4^), referenced values [13, 14] and journal publisher estimates^5^ lead us to estimate this value around 0.45.

The above estimates imply that, on average, every 100 submissions to a journal comprise 30 that are accepted after one or more rounds of peer review, 45 that are desk rejected, and 25 that are rejected after review. Thus, among submissions sent out for review, 55% (30 / (30 + 25) are ultimately accepted. That is, the articles published represent 55% of all reviewed submissions, indicating that 45% of submissions that were reviewed were rejected. These values are undoubtedly speculative, but they are consistent with Publons’ estimates.

Therefore, to estimate the number of peer reviews per year, we used Publons’ [9] formula:

1 $$ Nr\  of\ submis\mathrm{s}{ions}_{accepted}\times Average\  Nr\  of\ {reviews}_{accepted}+ Nr\  of\ {submissions}_{rejected}\times Average\  Nr\  of\ {reviews}_{rejected} $$

To obtain these values, we had to estimate the number of peer reviews performed for articles in 2020. For that, we used the numbers provided by the Dimensions portal (www.dimensions.ai). The free version as well as the subscription version of Dimensions currently provide separate numbers for articles, chapters, proceedings, preprints, monographs, and edited books. For the sake of simplicity, our estimate is confined to articles.

The total number of articles published in 2020 according to the Dimensions database is 4,701,988. Assuming that this sum reflects the 55% acceptance rate of reviewed submissions, the number of reviewed but rejected submissions (the 45% of all reviewed submissions) are estimated to be globally 4,701,988/55*45 = 3,847,081. Based on these calculations, the total number of peer reviews for submitted articles in 2020 is 4,701,988*3 + 3,847,081*2 = 21,800,126.

### Time spent on reviews

Several reports exist for the average time a reviewer spends when reviewing a manuscript. All of these are unfortunately based on subjective reports by reviewers rather than an objective measure. The only thing resembling an objective indication we found was in the Publons dashboard (Publons.com), which as of 6 Aug 2021 indicated that the average length of reviews in their database across all fields is approximately 390 words. This highlights that the average review likely has substantive content beyond a yes/no verdict, but this cannot be converted to a time estimate. A 2009 survey responded to by 3597 randomly selected reviewers indicated that the reported average time spent on the last review was 6 h [15], a 2016 survey reported that the median reviewing time is 5 h [9]. Another survey in 2008 found that the average reported time spent reviewing was 8.5 h [16]. To be noted, it is likely that the second round of reviews do not take as long as the first one. To be conservative (and considering the tendency of people to overestimate how much time they work), we will use 6 h as the average time reviewers spend on each review.

**Fig. 1:**
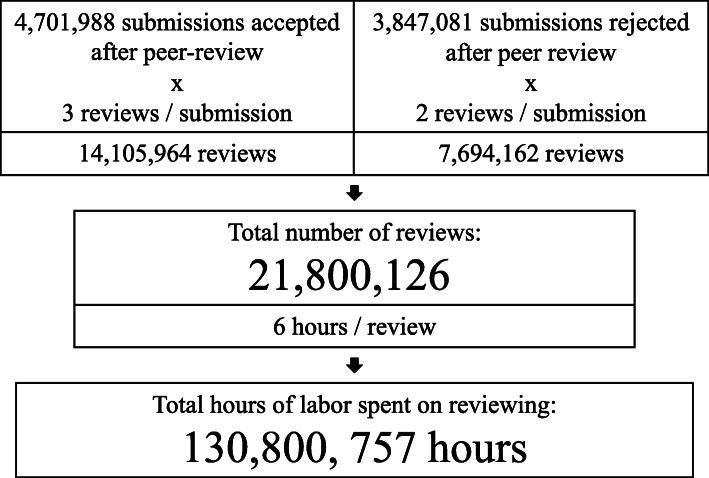
Overview of our calculation estimates of time spent on reviewing for scholarly articles in 2020. Number of published articles was obtained from Dimesions.AI database, all other numbers are assumptions informed by previous literature

Based on our estimate of the number of reviews and hours spent on a review, we estimate that in 2020 reviewers spent 21,800,126 × 6 h = 130,800,757 h on reviewing. This is equivalent to 14,932 years (at 365 days a year and 24 h of labor per day) (Fig. 1).

### Hourly wage of reviewers

To estimate the monetary value of the time reviewers spend on reviews, we multiplied reviewers’ average hourly wage by the time they spend reviewing. Note that some scholars consider their reviewing work to be volunteer work rather than part of their professional duties [5], but here we use their wages as an estimate of the value of this time. No data seem to have been reported about the wages of journal reviewers, therefore, we require some further assumptions. We assumed that the distribution of the countries in which reviewers work is similar to the distribution of the countries in the production of articles. In other words, researchers in countries that produce more articles also perform more reviews, while countries that produce few articles also do proportionally few reviews. Given the English-language and geographically Anglophone-centered concentration of scientific journals, we suspect that people in English-speaking countries are called on as reviewers perhaps even more than is their proportion as authors [17]. Because such countries have higher wages than most others, our assumption of reviewer countries being proportional to author countries is conservative for total cost. Accordingly, we calculated the country contributions to the global article production by summing the total number of publications for all countries as listed in the Dimensions database and computing the proportion of articles produced by each country.

Based on the results of the Peer Review Survey [15] and to keep the model simple and conservative, we assumed that reviewing is conducted almost entirely by people employed by academic workplaces such as universities and research institutes and that junior and senior researchers participate in reviewing in a ratio of 1:1. Therefore, to calculate the hourly reviewer wage in a given country we used.

2 $$ \frac{average\ annual\ post- doc\  salary+ average\ annual\ full\ professor\ salary}{2\times annual\ labor\ hours} $$

This yields a figure of $69.25 per hour for the U.S., $57.21 for the UK, and $33.26 for China (Table 1).

**Table 1:** Estimating the Value of Review Labor for the US, China, and the UK for 2020

Parameter	US	China	UK
Annual postdoc salary	$65,000	$68,174	$39,692
Annual professor salary	$179,736	$76,428	$116,731
Annual labor hours	1767	2174	1367
Reviewer hourly wage	$69.25	$33.26	$57.21
Articles	715,645	618,430	224,220
Contribution to global article production	16.68%	14.41%	5.22%
Reviews	3,636,031	3,141,908	1,139,106
Value of reviewing time	$1,510,810,944	$626,945,064	$391,036,638

*Note.* Salary values were collected from https://inomics.com/sites/default/files/2018-05/INOMICS%20Salary%20Report%202018.pdf for the USA and the UK, and were downloaded on 2021.09.09. from http://www.salaryexplorer.com/salary-survey.php?loc=44&loctype=1&job=50&jobtype=1#disabled for China. To estimate the average full professor salary, we calculated the average of the 39 professor categories available at salaryexplorer.com. To convert the average Chinese salary to USD, we used the 2020 average exchange rates (6.90) from CNY to USD based on https://www.macrotrends.net/2575/us-dollar-yuan-exchange-rate-historical-chart (The calculations are available at the projects’ OSF page). Note that we are concerned that the Chinese salaries may be inaccurate, based on anecdotal feedback we have received. For China, labor hours were found in https://ourworldindata.org/working-hours; for the USA and the UK they were retrieved from https://stats.oecd.org/Index.aspx?DataSetCode=ANHRS. The numbers of articles published in 2020 for each country are from the Dimensions database. To calculate the value of reviewing time, we used the non-rounded form of the hourly wages

### Value of reviewing labor

We estimated the value of reviewing by multiplying the calculated hourly reviewer wage in a country by the number of estimated reviews in that country and the time preparing one review. We calculated each country’s share from the global number of reviews by using the country’s proportional contribution to global production of articles. In this calculation, each article produced by international collaborations counts as one to each contributing country. This yielded that the monetary value of reviewing labor for the three countries that contributed to the most articles in 2020, is: $USD 1.5 billion for the U.S., $626 million for China, and $391 million for the UK (Table 1). An Excel file including the formula used for the estimation in the present paper with interchangeable parameters is available at the OSF page of the project https://osf.io/xk8tc/.

## Discussion

The high price of scientific publishing receives a lot of attention, but the focus is usually on journal subscription fees, article processing charges, and associated publisher costs such as typesetting, indexing, and manuscript tracking systems e.g., [1]. The cost of peer review is typically no included. Here, we found that the total time reviewers worked on peer reviews was over 130 million hours in 2020, equivalent to almost 15 thousand years. The estimated monetary value of the time US-based reviewers spent on writing reviews was over 1.5 billion USD in 2020. For China-based reviewers, the estimate is over 600 million USD, and for UK-based, close to 400 million USD. These are only rough estimates but they help our understanding of the enormous amount of work and time that researchers provide to the publication system. While predominantly reviewers do not get paid to conduct reviews, their time is likely paid for by universities and research institutes.

Without major reforms, it seems unlikely that reviewing will become more economical, relative to other costs associated with publishing. One reason is that while technology improvements may automate or partially automate some aspects of publishing, peer review likely cannot be automated as easily. However, reducing details that reviewers should check might soon become automated (see https://scicrunch.org/ASWG).

A second issue is that while there is much discussion of how to reduce other costs associated with publishing, little attention has been devoted to reducing the cost of peer review, even though it would likely be the costliest component of the system if reviewers were paid for the reviewers – rather than conducting the reviews under their “salary” paid time. After a long period of above-inflation subscription journal price increases, funders have attempted to put downward pressure on prices through initiatives such as *Plan S* [18] and through funding separate publishing infrastructures e.g., Wellcome Open Research and Gates Open Research [19, 20]. However, because publishers do not have to pay for peer review, putting pressure on publishers may have no effect on review labor costs. Peer review labor sticks out as a large cost that is not being addressed systematically by publishers. In another domain, research funders have worked on reducing the cost of paid grant review, for example by shortening the proposals or reducing the need for consensus meetings after individual assessments [21].

Here we will discuss two reforms to reduce the cost of peer review. The first would decrease the amount of labor needed per published article by reducing redundancy in reviews. The second would make better use of less-trained reviewers. Finally, we will briefly mention a few other reforms that may not reduce cost per review but would boost the benefits of peer review, thus improving the cost-benefit ratio.

### Reducing redundancy in peer review

Many manuscripts get reviewed at multiple journals, which is a major inefficiency e.g., [22]. Because this is a multiplicative factor, it exacerbates the issue of the rising global increase in number of submissions. While improvements in the manuscript between submissions means that the reviewing process is not entirely redundant, typically at least some of the assessment being done is duplication. Based on survey data [23], we conservatively estimated that, on average, a manuscript is submitted to two journals before acceptance (including the accepting journal). In other words, each accepted article has one rejection and resubmission behind it. Should the reviews of a previous submission be available to the journal of the new submission, reviewing time could be substantially reduced (presuming that the quality of review does not differ between journals – and it very likely does), but unfortunately this is not common practice. If we assume that the “passed on” or open reviews would reduce the requirements by one review per manuscript, then approx. 28 M hours (of our 85 M hour total estimate) could be saved annually. In the US alone, it would mean a savings of approx. 297 M USD of work.^6^

Some savings of this kind have already begun. Several publishers or journals share reviews across their own journals (PLOS, Nature [24]), which is sometimes known as “cascading peer review” [25]. Some journals openly publish the reviews they solicit (e.g., eLife; Meta-psychology; PLOS; Research Integrity and Peer Review; for a recent review see [26]), although typically not when the manuscript is rejected (Meta-psychology is an exception, and eLife will publish the reviews after a rejected manuscript is accepted somewhere else). The Review Commons initiative allows authors to have their preprint reviewed, with those reviews used by journal publishers including EMBO and PLoS [27]. Similarly, Peer Community In (peercommunityin.org) solicits reviews of preprints that can then be used by journals, including over 70 that have indicated they will consider such reviews.

A decline in the amount of research conducted, or the number of manuscripts this research results in, would reduce the amount of peer review labor needed. The number of articles being published has been growing rapidly for many decades [28, 29]. Some of this may be due to salami slicing (publishing thinner papers, but more of them), but this is not necessarily true - one study found that researchers’ individual publication rate has not increased [30] when normalized by the number of authors per paper, suggesting that authors are collaborating more to boost their publication count rather than publishing thinner papers. Hence, the increase in publication volume may be more a result of the steady increase in the global economy and, with it, support for researchers. Quality rather than publication quantity has, however, recently begun to be emphasized more by some funders and national evaluation schemes, and this may moderate the rate of growth in number of publications and potentially the peer review burden [31].

### Improving the allocation of review labor

#### Broadening and deepening the reviewer pool

Journal editors disproportionately request reviews from senior researchers, whose time is arguably the most valuable. One reason for this is that senior researchers on average show up more often in literature searches, and also editors favor people they are familiar with, and younger researchers have had less time to become familiar to editors [32]. With the same individuals tapped more and more, the proportion of requests that they can agree to falls [33], which is likely one reason that editors have to issue increasing numbers of requests to review (a contributor to increasing costs which we did not calculate). Journal peer review, therefore, takes longer and longer because the system fails to keep up with academia’s changing demographics [3]. Today, more women and minorities are doing academic research, and the contributions from countries such as China are growing rapidly. But many of these researchers don’t show up on the radar of the senior researchers, located disproportionately in North America and Europe, who edit journals. This can be addressed by various initiatives, such as appointing more diverse editors and encouraging junior researchers to sign up to databases that editors consult when they seek reviewers [34, 35].

A more substantial increase in efficiency might come from soliciting contributions to peer review from individuals with less expertise than traditionally has been expected. Journal editors traditionally look for world experts on a topic, whose labor is particularly costly in addition to being in short supply and in high demand. But perhaps contributions to peer review shouldn’t be confined only to those highly expert in a field. Evaluating a manuscript means considering multiple dimensions of the work and how it is presented. For some research areas, detailed checklists have been developed regarding all the information that should be reported in a manuscript (see www.equator-network.org). This provides a way to divide up the reviewing labor and have some aspects where even students, after some training, can vet aspects of manuscripts. Thus, we are hopeful that after more meta-research on what is desired from peer review for particular research areas, parts of peer review can be done by people who are not experts in the very specific topic of a manuscript but can nonetheless be very capable at evaluating particular aspects of a manuscript (and as mentioned above, automation can help with some tasks).

This process could also lead to greater specialization in peer review. For example, for manuscripts that report clinical trials, some people could be trained in evaluating the blinding protocol and resulting degree of success of blinding [36], and if they had the opportunity to evaluate that particular portion of many manuscripts, they grow better at it and thus can evaluate more in a shorter time, reducing the number of hours of labor that need be paid for. To some extent, this specialization in peer review has already begun. As reporting standards for particular kinds of research have become more widespread (e.g., Consolidated Standards of Reporting Trials (CONSORT) for clinical trials, Animal Research: Reporting of In Vivo Experiments (ARRIVE) for animal research, and Preferred Reporting Items for Systematic Reviews and Meta-analyses (PRISMA) for systematic reviews of randomized trials^7^), professional staff at some publishers have begun performing some checks for compliance with these standards. For example, staff at *PLOS* check all manuscripts on human subject research for a statement regarding compliance with the Declaration of Helsinki, and clinical trials research for a CONSORT statement. These staff presumably can do this job more efficiently, and do so for a lower salary, than an academic charged with peer reviewing every word of an entire manuscript. There are also some services (e.g., RIPETA,^8^ PUBSURE^9^) that automatically screen the to-be-submitted manuscripts and provide reports on potential errors and instant feedback to the authors, while other products (e.g., AuthorONE^10^) support publishers with automatic manuscripts screening including technical readiness checks, plagiarism checks, and checking for ethics statements.

### Unlocking the value of reviews

Some reforms to peer review would not reduce the cost per review, but would increase the benefits per review, improving the cost-benefit ratio. One such reform is making reviews public instead of confidential. Under the currently-dominant system of anonymised peer review, however, only the authors, other reviewers, and editor of the manuscript have the opportunity to benefit from the content of the review.

When reviews are published openly, the expert judgments and information within reviews can benefit others. One benefit is the judgments and comments made regarding the manuscript. Reviews often provide reasons for caution about certain interpretations, connections to other literature, points about the weaknesses of the study design, and what the study means from their particular perspective. While those comments influence the revision of the manuscript, often they either don’t come through as discrete points or the revisions are made to avoid difficult issues, so that they don’t need to be mentioned.

It is not uncommon for some of the points made in a review to also be applicable to other manuscripts. Some topics of research have common misconceptions that lead to certain mistakes or unfortunate choices in study design. Some of the experienced researchers that are typically called upon to do peer review can rapidly detect these issues, and pass on the “tips and tricks” that make for a rigorous study of a particular topic or that uses a particular technique. But because peer reviews are traditionally available only to the editor and authors of the reviewed study, this dissemination of knowledge happens only very slowly, much like the traditional apprenticeship system required for professions before the invention of the printing press. How much more productive would the scientific enterprise be if the information in peer reviews were unlocked? We should soon be able to get a better sense of this, as this is already being done by the journals that have begun publishing at least some of their peer reviews (e.g, *Meta-psychology, eLife, the PLOS journals; F1000Research, Royal Society Open Science, Annals of Anatomy, Nature Communications, PeerJ* [20]). It will be very difficult, however, to put a financial value on the benefits. Fortunately, there are also other reasons that suggest that such policies should be adopted, such as providing more information about the quality of published papers.

In some cases, performing a peer review can actually benefit the reviewer. In Publons’ 2018 reviewer survey, 33% of respondents indicated that one reason (they could choose two from a list of nine) they agreed to review manuscripts was to “Keep up-to-date with the latest research trends in my field.” (p12 9). If more of such people can be matched with a manuscript, reviewing becomes more of a “win-win”, with greater benefits accruing to the reviewer than may be typical in the current system. Better matching, then, would mean an increased return on the portion of an employer’s payment of a researcher’s salary that pays for peer review. The initiatives that broaden the reviewer pool beyond the usual senior researchers that editors are most likely to think of may have this effect.

### Limitations

A limitation of the present study is that it does not quantify academic editors’ labor, which is typically funded by universities, research institutes or publishers and is integral to the peer review process. At prestige journals with high rejection rates, a substantial proportion of (associate) editors’ time is spent desk-rejecting articles, which could be considered wasteful, as rejected articles are eventually published somewhere else. Which also requires additional work from authors to prepare the manuscripts and navigate different submission systems.

Additionally, our study’s limitations come from the poverty of the available data. For example, today, no available database covers all scholarly journals and their articles. The rates of acceptance and rejections we used are approximate estimates. The average time spent on reviews likely strongly depends on fields and length of manuscript and we do not know how representative the number we used is of all academia. We could not calculate the cost of review for journal articles and conference papers separately, although they might differ in this regard. The nationality and salary of the reviewers are not published either, therefore, our calculations need to be treated with caution as they have to rely on broad assumptions. Nevertheless, the aim of this study was to estimate only the magnitude of the cost of peer review without the ambition to arrive at precise figures. We encourage publishers and other stakeholders to explore and openly share more information about peer review activities to foster a fairer and more efficient academic world.

## Data Availability

The public dataset supporting the conclusions of this article is available from the https://app.dimensions.ai/discover/publication webpage. An Excel file including the formula used for the estimation in the present paper with interchangeable parameters is available at the OSF page of the project https://osf.io/xk8tc/.

## Footnotes

1 Based on Personal communication with the Publons team.

2 https://publons.com/researcher/?is_core_collection=1&is_last_twelve_months=1&order_by=num_reviews

3 Note, that there are cases when a single submitted review is prepared by more than one individual, but the used formula does not differentiate these cases from when a review is prepared by only one individual.

4 https://journals.plos.org/plosone/s/journal-information

5 https://www.elsevier.com/authors-update/story/publishing-tips/5-ways-you-can-ensure-your-manuscript-avoids-the-desk-reject-pile

6 (715,645 articles × 6 h)* $69.25

7 For their collection, see https://www.equator-network.org/.

8 https://ripeta.com/

9 https://pubsure.researcher.life/

10 https://www.enago.com/authorone-publisher.htm
